# Supplementary figures and images for: Forward genetic screen of homeostatic antibody levels in the Collaborative Cross identifies MBD1 as a novel regulator of B cell homeostasis
Source: PLoS Genet. 2022 Dec 27;18(12):e1010548. doi: 10.1371/journal.pgen.1010548 (PMC9829176; doi:10.1371/journal.pgen.1010548)

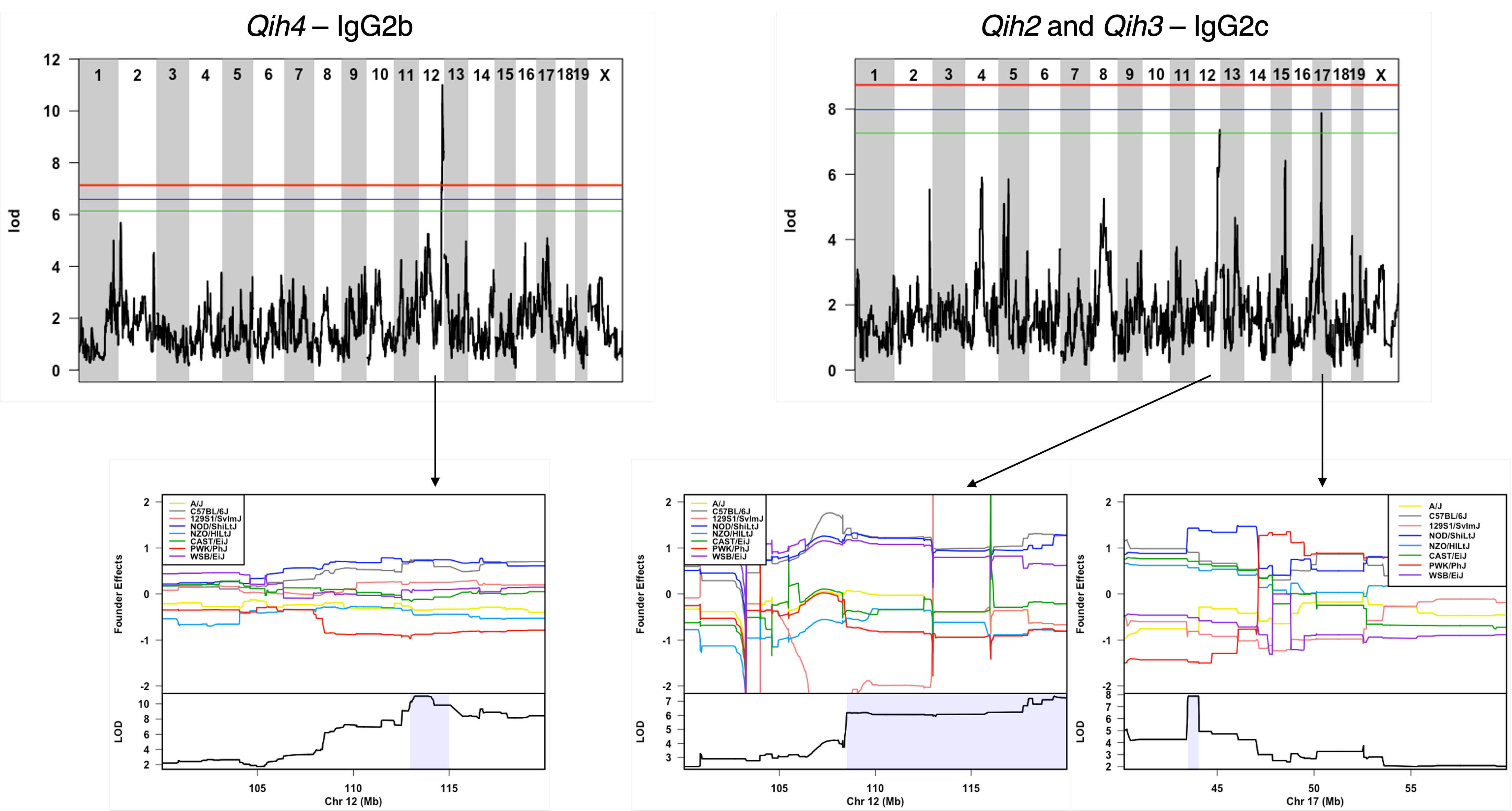

Supplement: S1 Fig — (TIFF) [file pgen.1010548.s001.tiff]

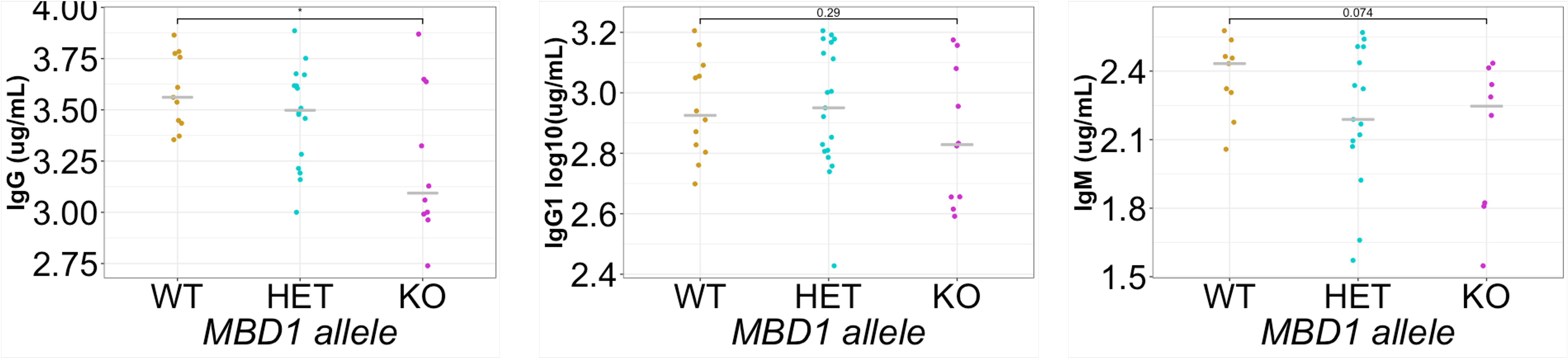

Supplement: S2 Fig — We assessed IgG1, Total IgG, and IgM levels in the serum of 15-16wk old animals. Each point represents an individual animal and the median for each genotype group on the x-axis is denoted by a crossbar. (*p < 0.05) (TIFF) [file pgen.1010548.s002.tiff]

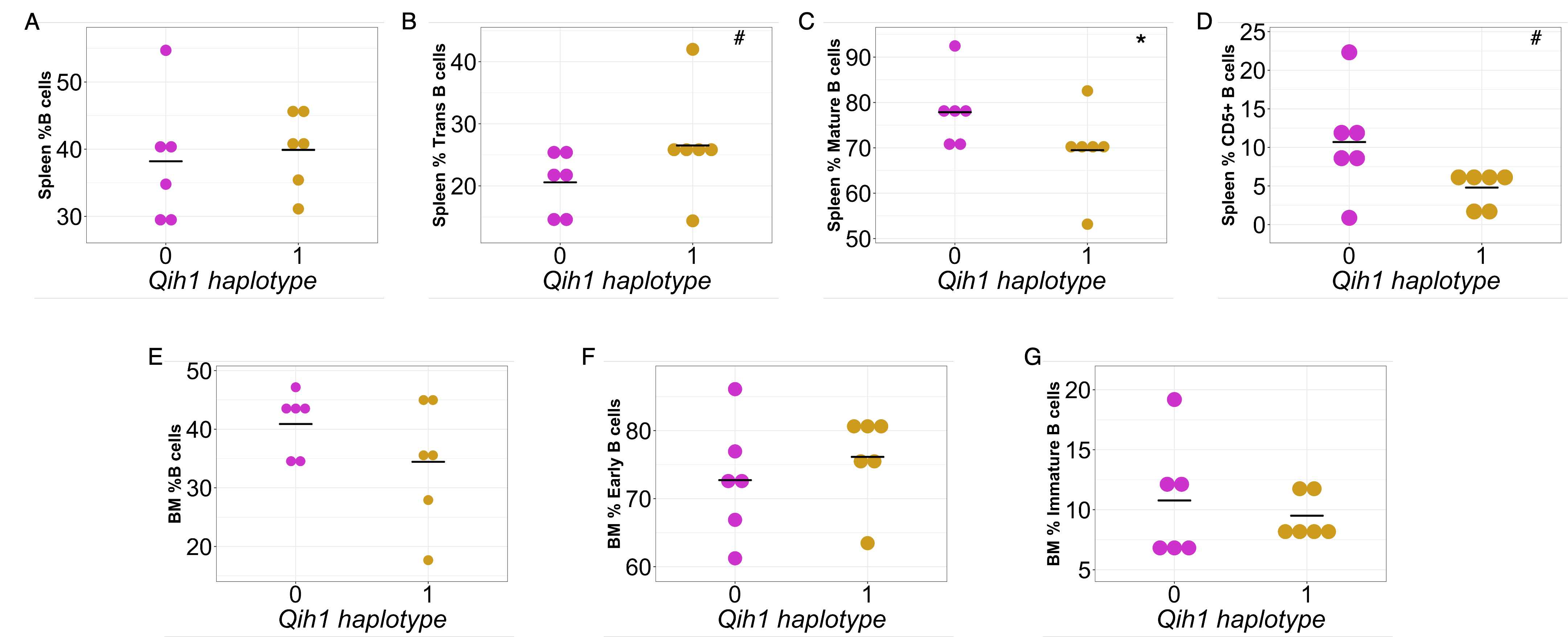

Supplement: S3 Fig — We assessed the relationship between B6, WSB, and CAST haplotypes (Qih1 haplotype = 1) and spleen total B220+ B cells (A), transitional (IgMvar, IgD-) B cells (B), mature (IgMvar, IgD+) B cells (C), B1 (CD5+) B cells (D), and bone marrow total B220+ B cells (E), early (IgM-, IgD-)B cells (F), and immature (IgM+, IgD-) B cells (G). Each point represents the mean value for each CC strain and the mean for each haplotype group on the x-axis is denoted by the grey crossbar. (#p < 0.2, *p < 0.1) (TIFF) [file pgen.1010548.s003.tiff]

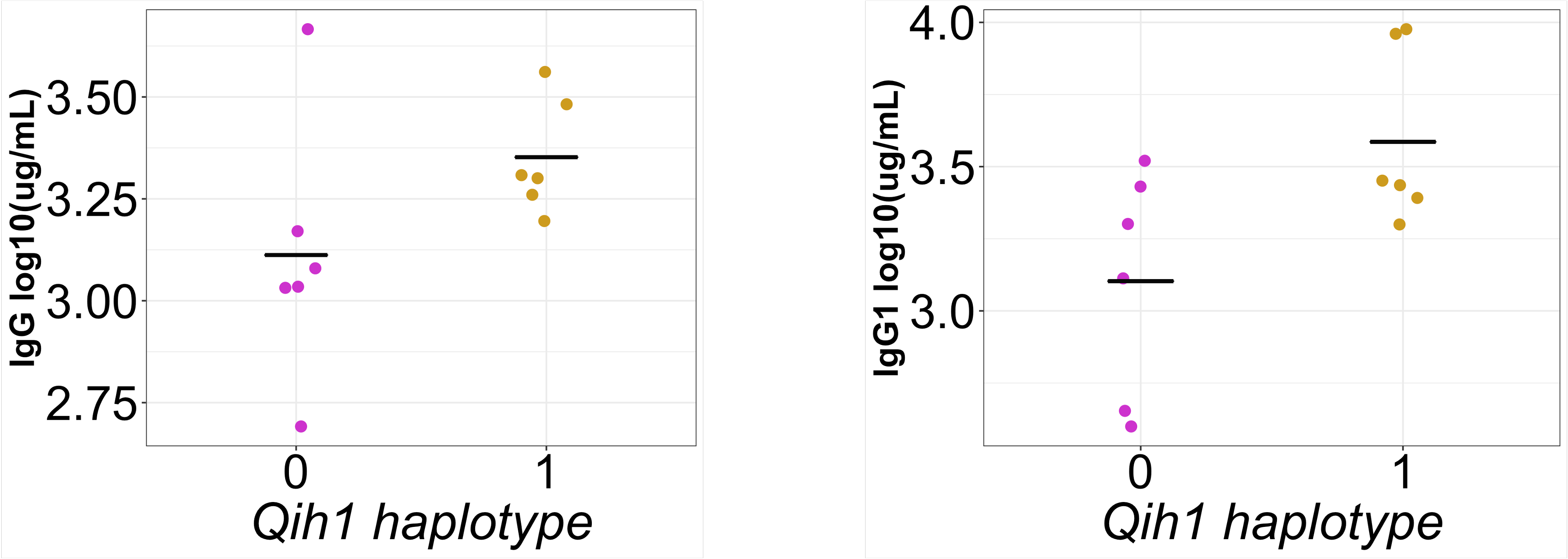

Supplement: S4 Fig — We assessed the relationship between B6, WSB, and CAST haplotypes (Qih1 haplotype = 1) and serum total igG and IgG1 levels. Each point represents the mean value for each CC strain and the mean for each haplotype group on the x-axis is denoted by the grey crossbar. (TIFF) [file pgen.1010548.s004.tiff]

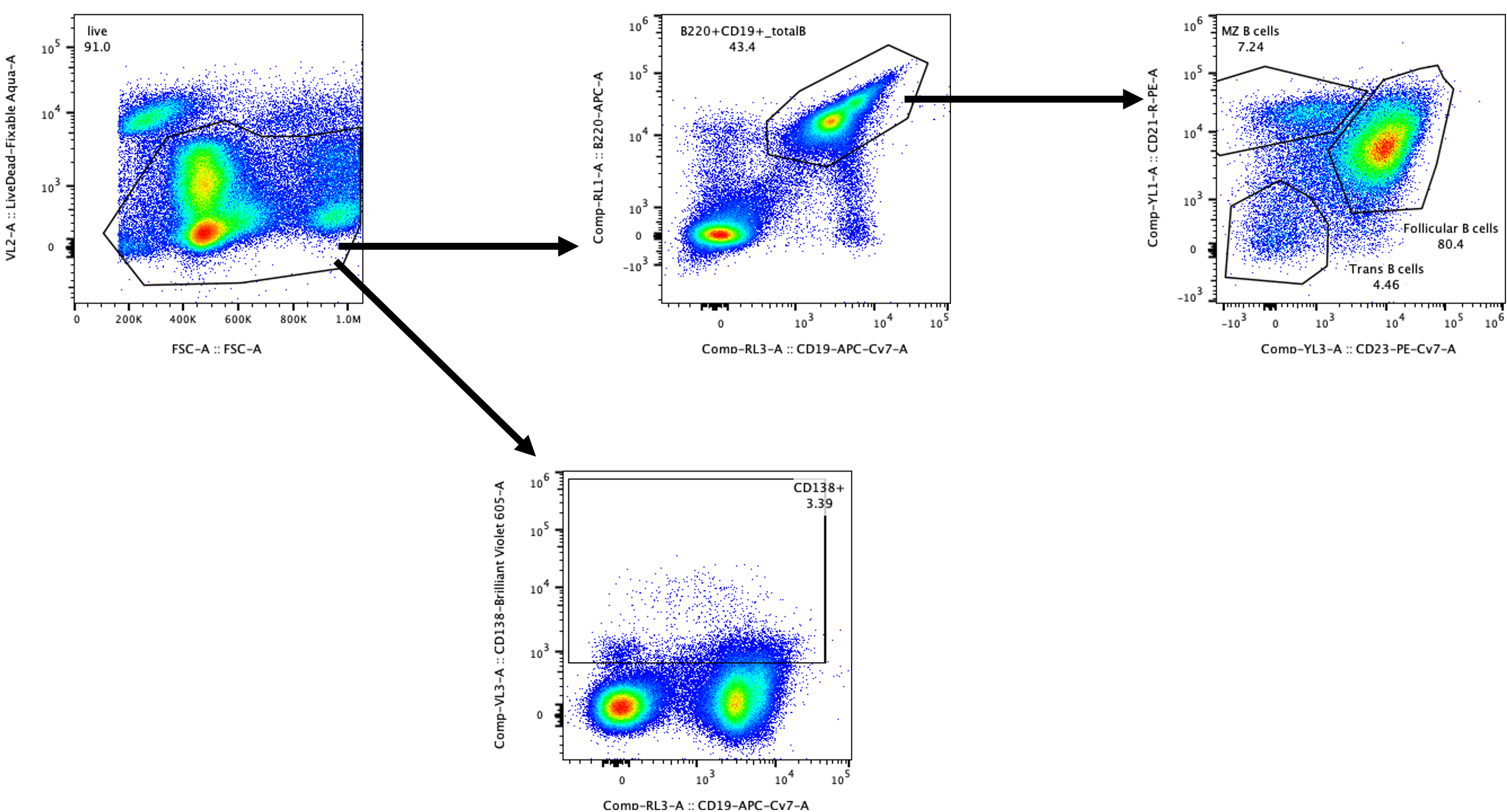

Supplement: S5 Fig — (TIFF) [file pgen.1010548.s005.tiff]
