# Supplementary material for: Forward genetic screen of homeostatic antibody levels in the Collaborative Cross identifies MBD1 as a novel regulator of B cell homeostasis
Source: PLoS Genet. 2022 Dec 27;18(12):e1010548. doi: 10.1371/journal.pgen.1010548 (PMC9829176; doi:10.1371/journal.pgen.1010548)
Supplement: S1 Table — P-values were determined using the cor.test function in R stats package (version 3.5.1). (DOCX) [file pgen.1010548.s006.docx]

S1 Table: p-values for antibody concentration correlations presented in Figure 1. P-values were determined using the cor.test function in R stats package (version 3.5.1).

|  | **Total IgG** | **IgG1** | **IgG2a** | **IgG2b** | **IgG2c** | **IgG3** | **IgM** | **IgA** |
| --- | --- | --- | --- | --- | --- | --- | --- | --- |
| **igg.cor** | **-** | 1.69E-22 | 1.50E-07 | 4.35E-19 | 0.00024198 | 2.66E-08 | 9.16E-05 | 0.01000014 |
| **igg1.cor** | 1.69E-22 | **-** | 0.00060463 | 1.96E-12 | 0.02342719 | 0.00015377 | 0.00109108 | 0.12239233 |
| **igg2a.cor** | 1.50E-07 | 0.00060463 | **-** | 3.45E-05 | 0.82580477 | 0.04018596 | 0.15634087 | 0.03638512 |
| **igg2b.cor** | 4.35E-19 | 1.96E-12 | 3.45E-05 | **-** | 3.66E-11 | 2.47E-08 | 0.00026604 | 0.00065344 |
| **igg2c.cor** | 0.00024198 | 0.02342719 | 0.82580477 | 3.66E-11 | **-** | 0.00034799 | 0.18141136 | 0.17766713 |
| **igg3.cor** | 2.66E-08 | 0.00015377 | 0.04018596 | 2.47E-08 | 0.00034799 | **-** | 1.76E-05 | 0.28644853 |
| **igm.cor** | 9.16E-05 | 0.00109108 | 0.15634087 | 0.00026604 | 0.18141136 | 1.76E-05 | **-** | 0.72241121 |
| **iga.cor** | 0.01000014 | 0.12239233 | 0.03638512 | 0.00065344 | 0.17766713 | 0.28644853 | 0.72241121 | **-** |
