# Supplementary material for: Forward genetic screen of homeostatic antibody levels in the Collaborative Cross identifies MBD1 as a novel regulator of B cell homeostasis
Source: PLoS Genet. 2022 Dec 27;18(12):e1010548. doi: 10.1371/journal.pgen.1010548 (PMC9829176; doi:10.1371/journal.pgen.1010548)
Supplement: S2 Table — (DOCX) [file pgen.1010548.s007.docx]

| S2 Table. Sequence evidence for alternative alleles for IgG2a and IgG2c | | | |
| --- | --- | --- | --- |
| CC Strain | Haplotype across Qih3 | IgG2c allele read counts^#^ | IgG2a allele read counts^#^ |
| CC010 | A/J | 0 | 38 |
| CC016 | A/J | 0 | 30 |
| CC022 | A/J | 0 | 47 |
| CC007 | C57BL/6J | 38 | 0 |
| CC030 | C57BL/6J | 41 | 0 |
| CC037 | C57BL/6J | 27 | 0 |
| CC006 | 129S1/SvImJ | 0 | 34 |
| CC013 | 129S1/SvImJ | 0 | 29 |
| CC027 | 129S1/SvImJ | 0 | 40 |
| CC031 | NOD/ShILtJ | 31 | 0 |
| CC049 | NOD/ShILtJ | 27 | 0 |
| CC057 | NOD/ShILtJ | 38 | 0 |
| CC024 | NZO/HlLtJ | 0 | 32 |
| CC045* | NZO/HlLtJ | 0 | 32 |
| CC051 | NZO/HlLtJ | 0 | 48 |
| CC003 | CAST/EiJ | 0 | 38 |
| CC012 | CAST/EiJ | 0 | 45 |
| CC021 | CAST/EiJ | 0 | 35 |
| CC008 | PWK/PhJ | 0 | 41 |
| CC035 | PWK/PhJ | 0 | 22 |
| CC046 | PWK/PhJ | 0 | 36 |
| CC004 | WSB/EiJ | 0 | 105 |
| CC015 | WSB/EiJ | 0 | 79 |
| CC023 | WSB/EiJ | 0 | 22 |

#NGS data from [1,2], which averaged 30x coverage across each strains genome, had their total reads queried for allele specific sequence (see Results).

*CC045 was not included in our phenotypic study of antibodies. We only had 2 strains homozygous for the NZO haplotype at the locus, and simply used this sequenced animal to confirm gene content.

**References**

1. Srivastava A, Morgan AP, Najarian ML, Sarsani VK, Sigmon JS, Shorter JS, et al. Genomes of the Mouse Collaborative Cross. Genetics. 2017;206(2):537-556. doi:10.1534/genetics.116.198838
2. Shorter JR, Najarian ML, Bell TA, Blanchard M, Ferris MT, Hock P, et al. Whole Genome Sequencing and Progress Toward Full Inbreeding of the Mouse Collaborative Cross Population. G3 (Bethesda). 2019;9(5):1303-1311. doi:10.1534/g3.119.400039
