# Supplementary material for: Forward genetic screen of homeostatic antibody levels in the Collaborative Cross identifies MBD1 as a novel regulator of B cell homeostasis
Source: PLoS Genet. 2022 Dec 27;18(12):e1010548. doi: 10.1371/journal.pgen.1010548 (PMC9829176; doi:10.1371/journal.pgen.1010548)
Supplement: S3 Table — (DOCX) [file pgen.1010548.s008.docx]

S3 Table: CC and inbred mouse strains used in these studies.

| Strains used in homeostatic antibody screen | Strains used in 48CC screen | Strains used in *Qih1* validation study | Strains used for MBD1 variant analysis |
| --- | --- | --- | --- |
| CC001/Unc | CC001/Unc | CC002/Unc | 129P2/OlaHsd |
| CC002/Unc | CC002/Unc | CC003/Unc | 129S1/SvImJ |
| CC003/Unc | CC003/Unc | CC004/TauUnc | 129S5SvEvBrd |
| CC004/TauUnc | CC004/TauUnc | CC008/GeniUnc | A/J |
| CC006/TauUnc | CC005 | CC013/GeniUnc | AKR/J |
| CC007/Unc | CC007/Unc | CC031/GeniUnc | BALB/cJ |
| CC008/GeniUnc | CC008/GeniUnc | CC032/GeniUnc | BTBR/T/Itpr3tf/J |
| CC009/Unc | CC011/Unc | CC037/TauUnc | BUB/BnJ |
| CC010/GeniUnc | CC013/GeniUnc | CC045/GeniUnc | C3H/HeH |
| CC011/Unc | CC015/Unc | CC058/Unc | C3H/HeJ |
| CC012/GeniUnc | CC016/GeniUnc | CC062/Unc | C57BL/10J |
| CC013/GeniUnc | CC017/Unc | CC074/Unc | C57BL/6NJ |
| CC015/Unc | CC019/TauUnc |  | C57BR/cdJ |
| CC016/GeniUnc | CC021/Unc |  | C57L/J |
| CC017/Unc | CC023/GeniUnc |  | C58/J |
| CC018/Unc | CC024/GeniUnc |  | CAST/EiJ |
| CC019/TauUnc | CC025/GeniUnc |  | CBA/J |
| CC021/Unc | CC026/GeniUnc |  | DBA/1J |
| CC022/GeniUnc | CC027/GeniUnc |  | DBA/2J |
| CC023/GeniUnc | CC029/Unc |  | FVB/NJ |
| CC024/GeniUnc | CC030/GeniUnc |  | I/LnJ |
| CC025/GeniUnc | CC031/GeniUnc |  | KK/HiJ |
| CC026/GeniUnc | CC032/GeniUnc |  | LEWES/EiJ |
| CC027/GeniUnc | CC033/GeniUnc |  | LP/J |
| CC028/GeniUnc | CC036/Unc |  | MOLF/EiJ |
| CC029/Unc | CC037/TauUnc |  | NOD/ShiLtJ |
| CC030/GeniUnc | CC038/GeniUnc |  | NZB/B1NJ |
| CC031/GeniUnc | CC039/Unc |  | NZO/HILtJ |
| CC032/GeniUnc | CC040/TauUnc |  | NZW/LacJ |
| CC033/GeniUnc | CC041/TauUnc |  | PWK/PhJ |
| CC035/Unc | CC042/TauUnc |  | RF/J |
| CC036/Unc | CC043/GeniUnc |  | SEA/GnJ |
| CC037/TauUnc | CC044/Unc |  | SPRET/EiJ |
| CC038/GeniUnc | CC045 |  | ST/bJ |
| CC039/Unc | CC046/Unc |  | WSB/EiJ |
| CC040/TauUnc | CC049/TauUnc |  | ZALENDE/EiJ |
| CC041/TauUnc | CC055/TauUnc |  |  |
| CC042/GeniUnc | CC057/Unc |  |  |
| CC043/GeniUnc | CC058/Unc |  |  |
| CC044/Unc | CC059/TauUnc |  |  |
| CC046/Unc | CC060/Unc |  |  |
| CC049/TauUnc | CC062/Unc |  |  |
| CC051/TauUnc | CC071/TauUnc |  |  |
| CC055/TauUnc | CC072/TauUnc |  |  |
| CC056/GeniUnc | CC078/Unc |  |  |
| CC057/Unc | CC080/Unc |  |  |
| CC058/Unc | CC081/Unc |  |  |
| CC059/TauUnc | CC082/Unc |  |  |
| CC060/Unc |  |  |  |
| CC061/GeniUnc |  |  |  |
| CC062/Unc |  |  |  |
| CC065/Unc |  |  |  |
| CC068/TauUnc |  |  |  |
| CC070/TauUnc |  |  |  |
| CC071/TauUnc |  |  |  |
| CC072/TauUnc |  |  |  |
| CC074/Unc |  |  |  |
| CC075/Unc |  |  |  |
