# Supplementary material for: Forward genetic screen of homeostatic antibody levels in the Collaborative Cross identifies MBD1 as a novel regulator of B cell homeostasis
Source: PLoS Genet. 2022 Dec 27;18(12):e1010548. doi: 10.1371/journal.pgen.1010548 (PMC9829176; doi:10.1371/journal.pgen.1010548)
Supplement: S4 Table — (DOCX) [file pgen.1010548.s009.docx]

S4 Table: Phenotype distribution and heritability of 48CC screen phenotypes.

| Phenotype | Phenotypic range | Median phenotype value | Broad sense heritability estimate |
| --- | --- | --- | --- |
| CD3^+^ T cells | 0.113 – 0.678 | 0.342 | 0.493 |
| CD4^+^ T cells  (% of CD3^+^ T cells) | 0.339 – 0.782 | 0.606 | 0.761 |
| CD8^+^ T cells  (% of CD3^+^ T cells) | 0.121 – 0.489 | 0.269 | 0.706 |
| CD19^+^ B cells | 0.246 – 0.622 | 0.441 | 0.243 |
| CD11b^+^ cells  (% of CD3^-^, CD19^-^) | 0.010 – 0.160 | 0.038 | 0.308 |
| CD11c^+^ cells  (% of CD3^-^, CD19^-^) | 0.007 – 0.968 | 0.022 | 0.428 |
