# Supplementary material for: Forward genetic screen of homeostatic antibody levels in the Collaborative Cross identifies MBD1 as a novel regulator of B cell homeostasis
Source: PLoS Genet. 2022 Dec 27;18(12):e1010548. doi: 10.1371/journal.pgen.1010548 (PMC9829176; doi:10.1371/journal.pgen.1010548)
Supplement: S5 Table — (DOCX) [file pgen.1010548.s010.docx]

|  | h^2^ | h^2^ lower | h^2^ upper |
| --- | --- | --- | --- |
| Total IgG | 0.6205634 | 0.6061534 | 0.6349734 |
| IgG1 | 0.6528361 | 0.6396505 | 0.6660217 |
| IgG2a | 0.6198290 | 0.5971154 | 0.6425426 |
| IgG2b | 0.7648681 | 0.7579627 | 0.7717736 |
| IgG2c | 0.8111844 | 0.7975761 | 0.8247927 |
| IgG3 | 0.3963995 | 0.3606554 | 0.4321435 |
| IgM | 0.7207755 | 0.7096106 | 0.7319403 |
| IgA | 0.6003100 | 0.5611781 | 0.6394419 |

Supplemental Table 5. Narrow sense heritability estimates for baseline serum antibody concentrations presented in figure 1 and table 1.
